# Supplementary material for: JAK Signaling Is Critically Important in Cytokine-Induced Viral Susceptibility of Keratinocytes
Source: Int J Mol Sci. 2023 May 25;24(11):9243. doi: 10.3390/ijms24119243 (PMC10252468; doi:10.3390/ijms24119243)
Supplement: Supplementary file 1 [file ijms-24-09243-s001.zip › ijms-2360792-supplementary.pptx]

## Slide 1
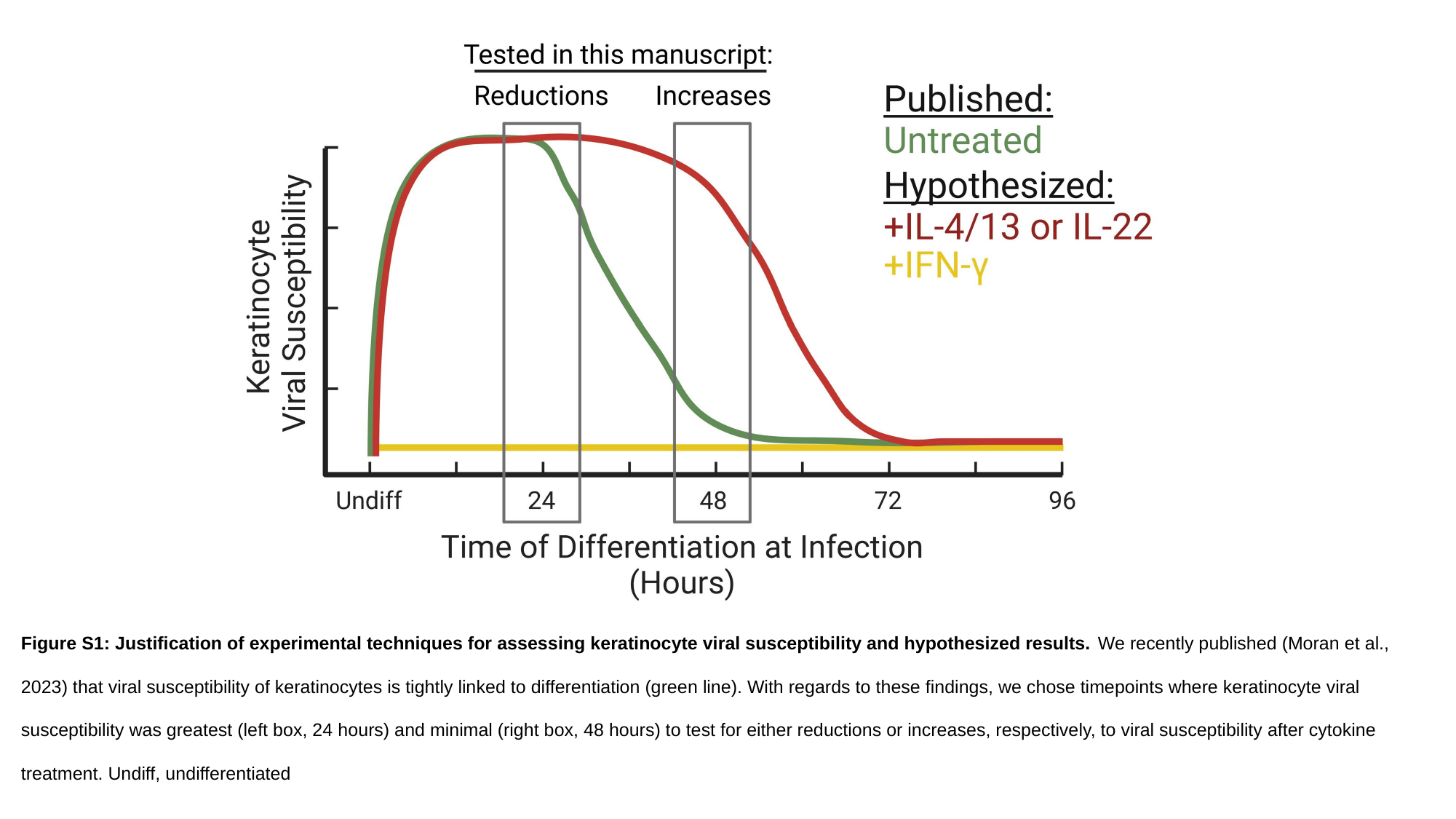

Figure S1: Justification of experimental techniques for assessing keratinocyte viral susceptibility and hypothesized results. We recently published (Moran et al., 2023) that viral susceptibility of keratinocytes is tightly linked to differentiation (green line). With regards to these findings, we chose timepoints where keratinocyte viral susceptibility was greatest (left box, 24 hours) and minimal (right box, 48 hours) to test for either reductions or increases, respectively, to viral susceptibility after cytokine treatment. Undiff, undifferentiated

## Slide 2
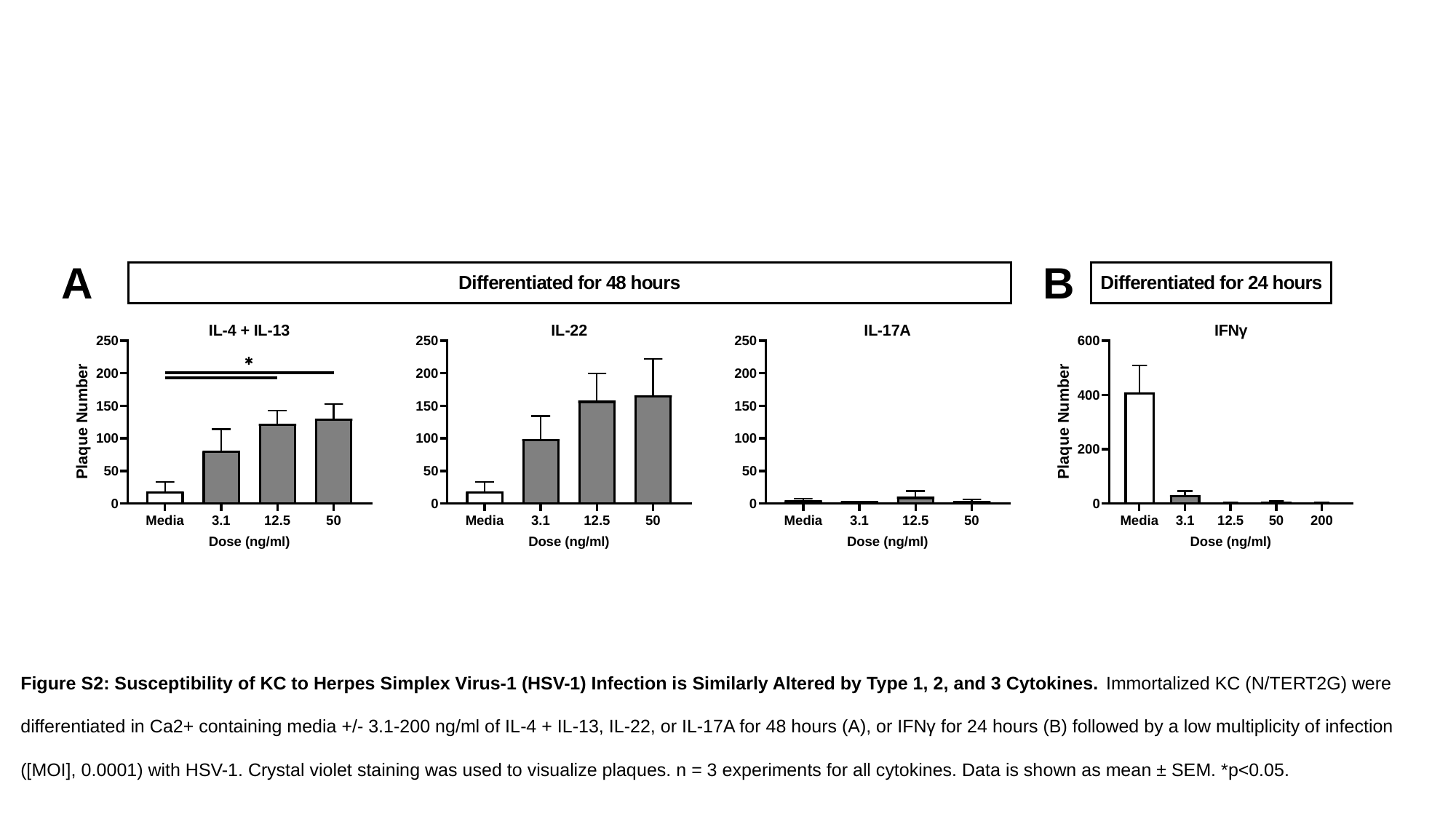

Figure S2: Susceptibility of KC to Herpes Simplex Virus-1 (HSV-1) Infection is Similarly Altered by Type 1, 2, and 3 Cytokines. Immortalized KC (N/TERT2G) were differentiated in Ca2+ containing media +/- 3.1-200 ng/ml of IL-4 + IL-13, IL-22, or IL-17A for 48 hours (A), or IFNγ for 24 hours (B) followed by a low multiplicity of infection ([MOI], 0.0001) with HSV-1. Crystal violet staining was used to visualize plaques. n = 3 experiments for all cytokines. Data is shown as mean ± SEM. *p<0.05.

## Slide 3
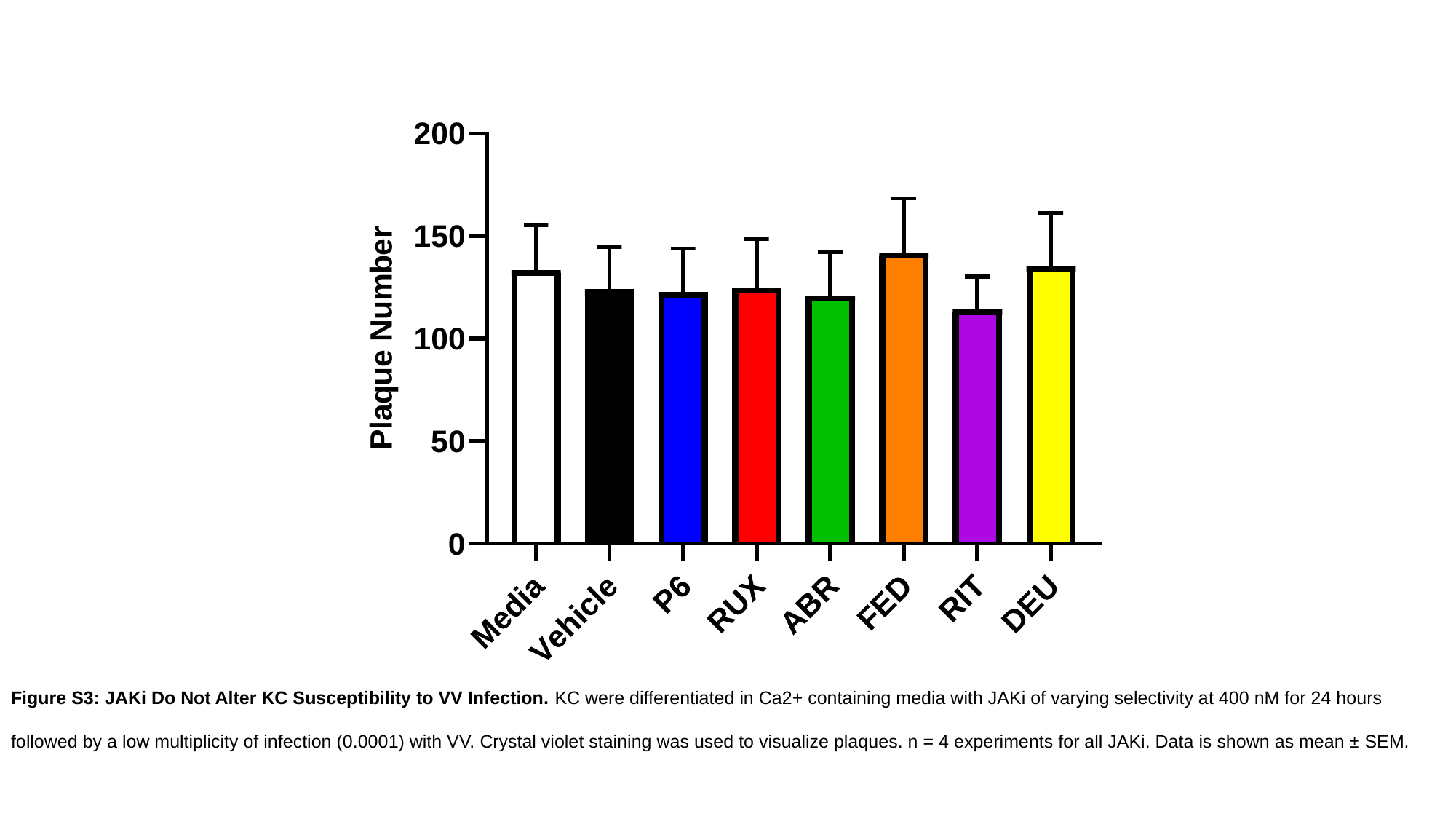

Figure S3: JAKi Do Not Alter KC Susceptibility to VV Infection. KC were differentiated in Ca2+ containing media with JAKi of varying selectivity at 400 nM for 24 hours followed by a low multiplicity of infection (0.0001) with VV. Crystal violet staining was used to visualize plaques. n = 4 experiments for all JAKi. Data is shown as mean ± SEM.

## Slide 4
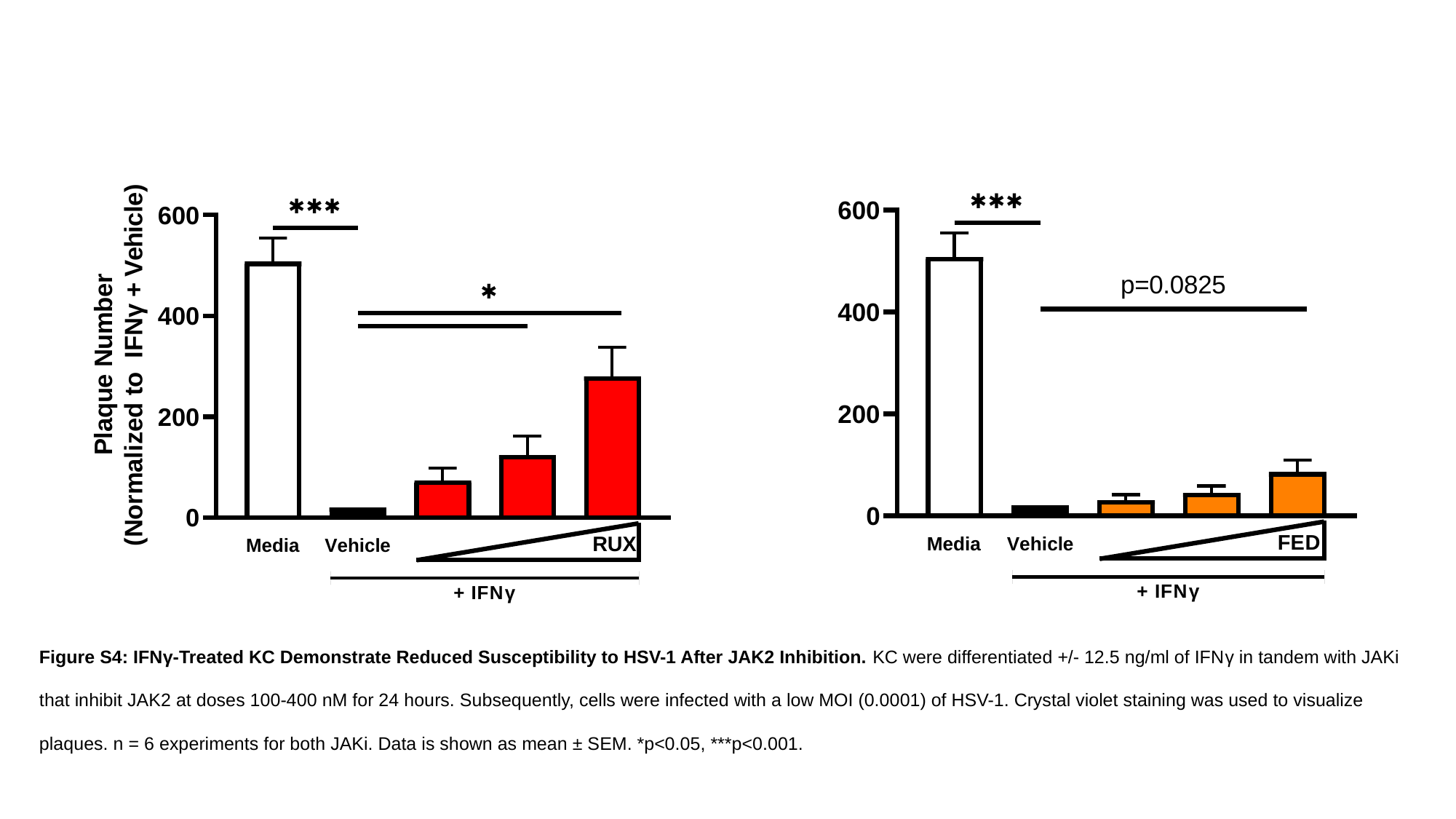

Figure S4: IFNγ-Treated KC Demonstrate Reduced Susceptibility to HSV-1 After JAK2 Inhibition. KC were differentiated +/- 12.5 ng/ml of IFNγ in tandem with JAKi that inhibit JAK2 at doses 100-400 nM for 24 hours. Subsequently, cells were infected with a low MOI (0.0001) of HSV-1. Crystal violet staining was used to visualize plaques. n = 6 experiments for both JAKi. Data is shown as mean ± SEM. *p<0.05, ***p<0.001.

## Slide 5
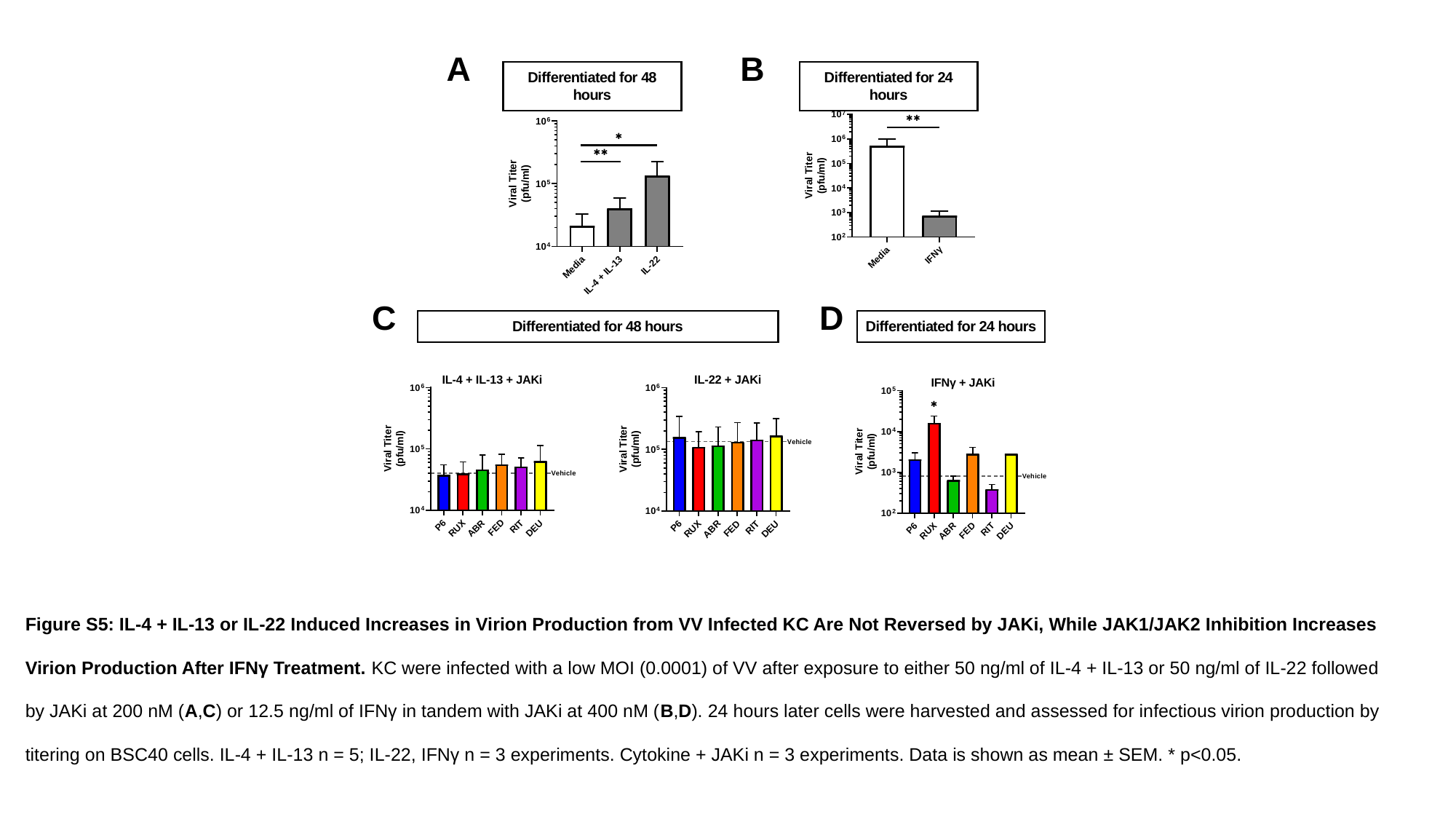

Figure S5: IL-4 + IL-13 or IL-22 Induced Increases in Virion Production from VV Infected KC Are Not Reversed by JAKi, While JAK1/JAK2 Inhibition Increases Virion Production After IFNγ Treatment. KC were infected with a low MOI (0.0001) of VV after exposure to either 50 ng/ml of IL-4 + IL-13 or 50 ng/ml of IL-22 followed by JAKi at 200 nM (A,C) or 12.5 ng/ml of IFNγ in tandem with JAKi at 400 nM (B,D). 24 hours later cells were harvested and assessed for infectious virion production by titering on BSC40 cells. IL-4 + IL-13 n = 5; IL-22, IFNγ n = 3 experiments. Cytokine + JAKi n = 3 experiments. Data is shown as mean ± SEM. * p<0.05.
